# Prévalence de l’hypotension orthostatique et ses facteurs favorisants chez les hypertendus noirs africains traités

**Published:** 2012-01-21

**Authors:** Soodougoua Baragou, Machiude Pio, Soulemane Pessinaba, Datouda Redah

**Affiliations:** 1 Service de Cardiologie, Centre Hospitalier Universitaire, Campus de Lomé, Togo

**Keywords:** Hypotension orthostatique, Hypertension artérielle, Afrique subsaharienne, Togo

## Abstract

**Introduction:**

Le but de cette étude était de déterminer la prévalence de l’hypotension orthostatique (HO) chez les hypertendus noirs africains traités, et rechercher ses facteurs favorisants.

**Méthodes:**

Il s’est agi d’une étude prospective transversale menée du 1^er^ février 2007 au 31 janvier 2009 à la clinique cardiologique du CHU Campus de Lomé, incluant des patients régulièrement traités pour hypertension artérielle. La pression artérielle et la fréquence cardiaque étaient mesurées en décubitus dorsal puis immédiatement en orthostatisme (1^ère^ et 3^ème^ minutes).

**Résultats:**

Sur une population de 394 patients hypertendus traités, 81 cas d’HO (20,5%) ont été observés dont 53 (65,4 %) étaient symptomatiques. Il s’agissait de 188 femmes (49,2%) et de 206 hommes (50,8%). L’âge moyen des hypertendus était de 53,4 ans ± 11,2 avec des extrêmes de 26 ans et de 81ans. Les patients ayant présenté une HO avaient un âge moyen significativement plus élevé : 60 ans contre 51 ans chez les autres (p= 0,01). L’HO était plus fréquente chez les patients traités par antihypertenseurs centraux, les patients ayant présenté un accident vasculaire cérébral, les diabétiques, les obèses.

**Conclusion:**

L’hypotension orthostatique est fréquente chez les hypertendus noirs africains traités. Il faut la rechercher systématiquement chez tous les hypertendus surtout mal contrôlés car elle peut être non seulement un facteur de mauvaise observance thérapeutique, mais aussi un facteur de risque cardiovasculaire indépendant.

## Introduction

L’hypotension orthostatique (HO) est la baisse de la pression artérielle systolique d’au moins 20 mmHg et 10 mmHg pour la diastolique, lors du passage en orthostatisme [1,2]. Elle est fréquente (14,6%) chez les hypertendus traités surtout âgés (>60 ans) et peut s’accompagner de symptômes gênants: malaises, vertiges, lipothymies et parfois syncopes [2–5]. Les causes de l’HO sont nombreuses, notamment les neuropathies, les endocrinopathies et aussi les effets secondaires des antihypertenseurs [6,7]. Les recommandations de la JNC VII [8] et la Société Européenne de Cardiologie [9] soulignent le contrôle insuffisant de l’hypertension artérielle chez les patients traités. Dans notre Service de Cardiologie au Centre Hospitalier (CHU) Campus de Lomé, le taux d’observance du traitement antihypertenseur est de 38% [10]. Les effets indésirables des médicaments peuvent être un facteur de mauvaise observance thérapeutique. De plus, l’HO est considérée comme un facteur de risque cardiovasculaire indépendant [11]. Nous avons entrepris ce travail pour évaluer la fréquence de l’HO chez l’hypertendu noir africain et en identifier les facteurs favorisants.

## Méthodes

Il s’est agi d’une étude prospective transversale menée du 1^er^ Février 2007 au 31 Janvier 2009 (2 ans) à la clinique cardiologique du CHU campus de Lomé, ayant concerné 394 patients hypertendus en traitement régulier (médicaments antihypertenseurs+mesures hygiéno-diététiques) depuis au moins 1 mois et bien observants. Nous avons exclu de l’étude, les patients traités par d’autres médicaments pouvant entraîner une hypotension artérielle (dérivés nitrés, psychotropes). Un tensiomètre électronique validé, de marque Omron Inc, avec un brassard adapté aux bras des patients était utilisé. La pression artérielle et la fréquence cardiaque ont été mesurées en décubitus dorsal, puis à une minute et trois minutes après le passage en orthostatisme. Une symptomatologie fonctionnelle en rapport avec une hypotension orthostatique a été recherchée. D’autres facteurs de risque cardiovasculaire (obésité, diabète) et les pathologies associées (insuffisance cardiaque, infarctus du myocarde, accident vasculaire cérébral) ont été colligés. La taille des patients a été mesurée à l’aide d’une toise, le poids à l’aide d’une balance de marque Seca. L’obésité était définie par un indice de masse corporelle (IMC):poids en Kg/(taille en m)^2^ ≥ 30 Kg/m^2^. Le diabète était défini par une glycémie à jeun >1,26 g/ (ou 7mmol/l) à deux dosages consécutifs espacées de 2 semaines, ou>2 g/l (ou 11 mmol/l) dès le premier dosage. L’hypotension orthostatique a été affirmée lorsque nous avons constaté une baisse de la pression artérielle systolique d’au moins 20 mmHg et/ou une baisse de la pression artérielle diastolique d’au moins 10 mmHg, même en l’absence de symptomatologie fonctionnelle. L’analyse des données, effectuée avec le logiciel Epi Info 6.0, a été descriptive et parfois comparative avec un seuil de signification de 0,05.

## Résultats

### Caractéristiques cliniques et thérapeutiques générales de notre échantillon d’étude

Il était composé de 394 patients hypertendus traités dont 188 femmes (49,2%) et de 206 hommes (50,8%). L’âge moyen était de 53,4 ans±11,2 avec des extrêmes de 26 ans et de 81 ans. Seulement 10,9% de nos patients (n= 43) avaient une assurance maladie ou couverture sociale. Les sujets âgés de plus de 60 ans représentaient 41,3% (n= 163) de notre échantillon. L’hypertension artérielle n’était contrôlée (TA<140/90 mmHg) que dans 36,5% des cas (n=144). La prévalence de l’obésité était de 16% (n= 63), et celle du diabète était de 13,7% (n= 54). Soixante-dix pour cent (n= 38) des diabétiques souffraient de neuropathie diabétique. Trente-sept patients (soit 9,4%) avaient un antécédent d’accident vasculaire cérébral. Lors du passage en orthostatisme, une mauvaise adaptation de la fréquence cardiaque (fréquence cardiaque invariable ou abaissée) était observée chez 27,7% des patients (n= 109). Les antihypertenseurs les plus utilisés étaient les diurétiques (73.8%), les inhibiteurs de l’enzyme de conversion (39%) et les inhibiteurs calciques (34%). Les centraux et les bêtabloquants étaient prescrits respectivement dans 17,2% et 14,4% des cas. Plus de la moitié des patients (51,3%; n=202) était traitée par au moins deux antihypertenseurs (Table 1).

**Table 1:** Fréquences de l’hypotension orthostatique en fonction des classes thérapeutiques utilisées en monothérapie et en associations et des autres pathologies associées chez les hypertendus

Classes thérapeutiques	Patients		Hypotension orthostatique	
	
	n	%	n	%
Diurétiques*	63	16	7	11,1
IEC	47	12	4	8,5
IC	36	9,1	3	8,3
Bêtabloquants	13	3,3	3	23
Centraux	25	6,3	9	76
ARAII	8	2	00	00
Diurétique*+IEC	40	10,1	4	10
Diurétique*+IC	32	8,1	3	9,3
Diurétique*+Bêtabloquant	11	2,8	3	27,2
Diurétique*+ARA II	10	2,5	1	10
Diurétique*+Centraux	9	2,3	6	66,6
IEC+IC	17	4,3	2	11,7
IEC+Bêtabloquant	13	3,3	2	15
IEC+Centraux	21	5,3	10	47
IC+Bêtabloquant	14	3,5	2	14,3
IC+ARA II	5	1,3	0	0
IC+Centraux	6	1,5	4	66,6
Diurétique*+IEC+IC	11	2,8	2	18
Diurétique*+Bêtabloquant+IC	6	1,5	1	16,6
Diurétique*+IC+IEC+Centraux	7	1,8	5	71,4
Pathologies associées				
HTA isolée	178	45,2	22	12,3
Accident vasculaire cérébral	37	9,4	12	32,4
Insuffisance cardiaque	50	12,7	9	18
Obésité	63	16	18	30
Diabète	54	13,7	20	37
Infarctus du myocarde	12	3	12	0

* Diurétiques thiazidiques et apparentés; ARA II : Antagonistes des Récepteurs de l’Angiotensine II IEC : Inhibiteurs de l’Enzyme de Conversion; IC : Inhibiteurs Calciques

### Prévalence de l’hypotension artérielle orthostatique et ses facteurs favorisants

Quatre-vingt-un cas d’HO (20,5%) ont été observés dont 53 (65,4%) étaient symptomatiques: vertiges (58%), troubles visuels (30%) et lipothymies (11%). La prévalence de l’HO s’élevait à 32% chez les sujets de plus de 60 ans, sans différence significative par rapport au sexe (p>0,05). Les patients ayant présenté une HO avaient un âge moyen significativement plus élevé : 62±9 ans contre 51±10 ans chez les autres (p= 0,01). L’HO était plus fréquente (40%) chez les patients dont la fréquence cardiaque n’avait pas changé ou avait baissé lors du passage en orthostatisme par rapport à ceux qui avaient augmenté leur fréquence cardiaque (10,3%) p<0,001. Tous les cas d’HO étaient observés chez les patients dont l’HTA n’était pas contrôlée (TA ≥140/90 mmHg). En monothérapie, l’HO était plus fréquente (76%) chez les patients traités par antihypertenseurs centraux, suivis des diurétiques thiazidiques et des bêtabloquants. La fréquence de l’HO était également élevée avec les associations médicamenteuses comportant les antihypertenseurs centraux, et à un moindre degré les diurétiques thiazidiques et les bêtabloquants (Table 1). En fonction de la pathologie associée, l’HO était plus fréquente en cas de diabète, d’antécédent d’accident vasculaire cérébral (AVC), et d’obésité (Table 1).

## Discussion

Cette étude prospective a permis de mettre en évidence une prévalence élevée de l’HO particulièrement chez les sujets âgés de plus de 60 ans hypertendus traités. Dans des études occidentales, cette prévalence varie de 8 à 28% selon les auteurs et les populations étudiées, chez des hypertendus traités âgés d’au moins 65 ans [3,4,9,12]. Nous avons observé que 65,4% de ces HO étaient symptomatiques; cette symptomatologie gênante peut être à l’origine d’une mauvaise observance thérapeutique. Tous les cas d’HO ont été observés chez des patients (63,5%) dont l’HTA n’était pas contrôlée (TA>140/90 mmHg) sous traitement. Le contrôle insuffisant de l’HTA représente donc un facteur de risque d’HO [3,4,9,12]. Notre étude a confirmé le risque élevé d’hypotension artérielle orthostatique avec les antihypertenseurs centraux [6,7,12]. Ce risque était modéré dans notre série avec les diurétiques et les bêtabloquants, faible avec les autres antihypertenseurs. Ces résultats sont presqu’identiques à ceux de Kamaruzzaman et al [11] qui ont démontré en plus que la trithérapie et la quadrithérapie antihypertensives étaient fortement prédictives d’HO.

En Afrique subsaraharienne en général, et au Togo en particulier, les antihypertenseurs centraux (surtout l’alpha méthyldopa) sont encore prescrits à cause de leurs coût très abordable pour beaucoup de patients qui n’ont ni couverture sociale ni assurance maladie. Par contre, les antagonistes des récepteurs AT1 de l’angiotensine II (ARA II) sont peu utilisés à cause de leur coût élevé. Dans l’étude de Gonzalez et al [4], l’HO était plutôt associée aux bêtabloquants et aux inhibiteurs calciques non dihydropiridiques. Une donnée préoccupante est que l’HO est un facteur de risque d’accident vasculaire cérébral et d’accident coronarien [11,13–15]. En effet, selon les études de Rose et al.[11,14], l’HO multiplie le risque d’accident coronarien par 3,49 et de mortalité cardiovasculaire par 2,4. Notre étude confirme également le rôle de l’âge qui serait une des principales causes d’HO [2–4,6,7]. Le mécanisme essentiel serait une moins grande sensibilité des barorécepteurs mais aussi, des facteurs associés, en particulier une déplétion hydrosodée et l’absence d’augmentation des catécholamines lors de l’orthostatisme [6,7].

Nous avons observé une plus grande fréquence de l’HO chez les diabétiques, qui serait en rapport avec la neuropathie diabétique [6,7]. L’HO était aussi fréquente (32,1%) chez les patients ayant des antécédents d’accident vasculaire cérébral (AVC). Ceci confirme le rôle étiologique de l’AVC dans l’HO. Kong et al [15] ont trouvé une incidence d’HO de 52,1% au cours d’un suivi de 71 patients souffrant d’AVC et en rééducation fonctionnelle. Cette fréquence élevée de l’HO au cours des AVC s’explique essentiellement par la dysfonction des barorécepteurs et l’absence ou l’insuffisance de la contractilité des muscles des membres inférieurs (à cause de la paralysie) pour assurer un bon retour veineux en orthostatisme. Un cercle vicieux s’installe car l’HO à son tour peut aggraver l’AVC ischémique avec récidive d’AVC, prolongement du délai de récupération voire augmentation de la mortalité [11,15,16]. La fréquence accrue de l’HO chez le sujet obèse que nous avons observée n’est pas particulièrement décrite dans la littérature. Dans tous les cas, nos patients avaient des facteurs prédisposant qui s’intriquent. Les patients qui n’ont pas augmenté leur fréquence cardiaque lors du passage en orthostatisme ont présenté plus d’HO, évoquant donc une dysautonomie. En effet, une HO associée à une augmentation de la fréquence cardiaque évoque une hypovolémie, alors que l’absence d’une modification de la fréquence cardiaque évoque une dysautonomie par lésion organique de l’arc baroréflexe [5,6].

## Conclusion

L’hypotension orthostatique est fréquente chez les hypertendus noirs traités. Etant considérée comme un facteur de risque cardiovasculaire indépendant, et un facteur de mauvaise observance thérapeutique, elle doit être recherchée systématiquement, en particulier dans l’HTA non contrôlée, chez les sujets âgés, les diabétiques, les obèses, les patients sous antihypertenseurs centraux et chez ceux ayant un antécédent neurologique.

## Confit d’intérêt

Les auteurs ne déclarent aucun conflit d’intérêt.
